# Long-term effects of grandparental child neglect on adult grandchildren's mental health: *A three-generation study*

**DOI:** 10.1016/j.ssmph.2024.101712

**Published:** 2024-09-24

**Authors:** Emre Sarı, Mikko Moilanen, Maarten Lindeboom

**Affiliations:** a School of Business and Economics, UiT the Arctic University of Norway, Tromsø, Norway; b Division for Health and Social Sciences, NORCE Norwegian Research Centre, Oslo, Norway; c School of Business and Economics, Vrije Universiteit Amsterdam, Amsterdam, Netherlands; d Centre for Health Economics, Monash University, Melbourne, Australia

**Keywords:** Child neglect, Adverse childhood experiences, Childhood trauma, Mental health, Depression, Grandparental influence

## Abstract

Child neglect is a significant social problem with severe consequences for individuals and society. This study explores how intergenerational transmission of grandparental child neglect affects grandchildren's mental health in adulthood. We utilize a three-generational dataset from the Tromsø Study and estimate a linear probability model to find the distinct roles of both maternal and paternal grandparents. We test the additive risk hypothesis for continuous, intergenerational effects of child neglect in both the maternal and paternal lineages. Furthermore, we use structural equation modeling to test how sequential exposures to neglect across generations ultimately bear on adult mental health outcomes. Our results confirm the additive risk hypothesis but only for maternal grandparents: our findings show that only maternal parents' neglectful parenting is associated with an increased probability of depression in their grandchildren, conditional on whether their parents neglected them. These results contribute to research on intergenerational transmission by the finding that additive risks of child maltreatment flow down generations mainly through maternal lineages.

## Highlights

• Revealed maternal grandparents' key role in intergenerational child maltreatment.
• Confirms additive risk hypothesis, especially via maternal neglect.
• Expands beyond parental effects to underscore grandparental effect on mental health.
• Suggests considering grandparental roles in child neglect prevention policies.

## 1 Introduction

Child neglect is a form of child maltreatment, a significant global problem that violates children's right to a healthy and violence-free life and affects their mental, emotional, and physical well-being in adulthood, as well as the well-being of society as a whole (World Health Organization, 2006). Neglect in childhood is a common factor in the long-term development of adult mental health problems, particularly depression and anxiety, which have personal and costly societal consequences. The prevalence of the two aforementioned mental health problems is rapidly increasing worldwide (IHME, 2020), and they are the most frequently observed general mental health disorders in primary healthcare services in Norway (Norwegian Institute of Public Health, 2018). Given the overall heavy burden on individuals, depression and anxiety disorders also have a substantial effect on health systems economically and on society in general (Morrissey & Kinderman, 2020). Knowledge of the underlying mechanisms of these disorders is thus relevant for the formulation of appropriate preventive measures and the design of cost-effective policies (Persson & Rossin-Slater, 2018).

In this study, we examine the intergenerational transmission of child neglect and the resulting risk of depression, one of the most common mental health problems. Our research focuses on the relationship between grandparental neglect and grandchildren's mental health in adulthood. Recognizing the increasingly significant role that grandparents play in the lives of their grandchildren—a dynamic highlighted by Sari (2023) as an essential factor in the broader demographic and familial contexts—we explore how grandparental child neglect is associated with mental health issues in grandchildren, now adults over 40. We also analyze the distinct roles of maternal and paternal grandparents in relation to these adult grandchildren's mental health outcomes.

Two recent studies have addressed similar issues. Islam et al. (2023) examine the effects of breaking the cycle of child maltreatment across generations, and Su et al. (2022) review the intergenerational effects of maternal child maltreatment on offspring psychopathology through a meta-analysis of twelve studies. Our research goes beyond previous studies and makes valuable contributions. This study takes a more comprehensive approach than Islam et al. (2023) by examining the additive risk underlying the continuity of child neglect across generations. Our three-generation study also differs from previous research that relied mainly on the relationship between parents' adverse childhood experiences (see, e.g., Cooke et al., 2019, 2021; Seteanu & Giosan, 2022) and their children's mental health or the continuity of maltreatment across two generations (see, e.g., Armfield et al., 2021; Capaldi et al., 2019; Widom et al., 2015). Our approach focuses on the long-lasting effects of child neglect by studying whether these effects differ between neglectful maternal and paternal grandparents. Thus, while the literature focuses primarily on the intergenerational transmission between parents and their maternal grandparents (see, e.g., Johnston et al., 2013; Lotto et al., 2021; Yang et al., 2018), our study broadens the scope to include the lineage of both maternal and paternal grandparents.

Why is Norway an important country to study the effects of childhood trauma across multiple generations? Despite its reputation as a welfare state with good population health, significant health inequalities persist in Norway, as Mackenbach (2017) argued, with differences among the largest in Europe. Most previous research on health in Norway has focused on the life course of one generation and its mental health outcomes (see, e.g., Broekhof et al., 2022; Haugland et al., 2021). Limited research has considered intergenerational perspectives (Grönqvist et al., 2017; Myhre et al., 2014). These studies suggest that early life experiences are crucial in shaping long-term health outcomes and that significant health inequalities may persist even in developed countries with strong social welfare systems. Our study is the first to examine the effects of childhood trauma across three generations in Norway, adding to the important body of research on health inequalities in the country.

Our main results show that child neglect increases the probability of mental health problems in adulthood. Additionally, the effect of neglectful parenting on grandchildren is amplified when the maternal grandparents are also neglectful. Overall, our study adds to the literature on the intergenerational transmission of child neglect and highlights the importance of addressing this issue to improve the well-being of future generations.

### 1.1 Child neglect

Child maltreatment is a broad term that covers various types of abuse, emotional and physical mistreatment, and neglect (World Health Organization, 2006). Neglect, however, is the most common form of maltreatment; it is defined as the failure to meet a child's basic needs regarding nutrition, housing, clothing, healthcare, and supervision (Fallon et al., 2020; Yang et al., 2018). Therefore, poverty and parenting characteristics are often associated with child neglect, which has serious long-term consequences for children's physical and mental health (Slack et al., 2004). Whether it can be intentional or unintentional, it has serious long-term consequences for children's physical and mental health. In our study, “child neglect” aligns with the above definition and is explained in more detail in the Data section regarding the instructions for participants.

In Norwegian, the term for child neglect is *omsorgssvikt*, which has a heavy connotation due to Norway's long-standing commitment to child rights, rooted in historical developments such as the 1953 Child Protection Act. This act and subsequent legislation emphasized the child's best interest, making neglect a serious legal and social issue. Furthermore, under Norwegian law,^1^ the childcare service is responsible for instituting early measures to prevent serious neglect cases. If a child is found to be neglected, the child's family faces serious legal consequences. Therefore, we must emphasize the gravity of the situation since the issue of child neglect is addressed head-on as *omsorgssvikt* rather than only indirectly, as in the Tromsø Study.

### 1.2 Intergenerational transmission of child neglect

Neglectful behavior of parents toward their children is associated with variations in mental health outcomes across generations. This phenomenon has been observed in the literature and can be explained by various theories. However, we still do not comprehend its underlying mechanisms, which is of particular concern. Alink et al. (2019) noted that a better understanding of the mechanisms behind the intergenerational transmission of child neglect is urgently needed to develop effective interventions to prevent maltreatment in subsequent generations.

The intergenerational transmission of child neglect has been attributed to the intergenerational cycle of violence hypothesis (Abramovaite et al., 2015). According to this hypothesis, maltreatment in his/her childhood may result in aggression directly for an individual by increasing symptoms in their mental health or indirectly through enhanced emotional dysregulation (Brennan et al., 2021; Newton, 2019). It can also result in a cycle of abuse and neglect that can be perpetuated without any genetic link (Abramovaite et al., 2015). Langevin et al. (2023) study found that maternal emotional dysregulation and mother-to-child attachment are factors in the intergenerational continuity of child maltreatment. Widom (2017) study also showed that childhood neglect leads to the intergenerational transmission of abuse and neglect through the parent–child relationship. These findings implicate grandparents' neglect of their own children in the intergenerational transmission of child maltreatment and its associated mental health outcomes, such as depression, in adult grandchildren.

The most important of the many causal implications that child neglect has belongs to intergenerational transmission, adversity, and risk. This simply means that children who have been from a background of neglect are more likely to continue the cycle of neglect as adults, becoming either the neglectful parents themselves or becoming victims in a relationship of neglect (Bartlett et al., 2017; Berlin et al., 2011; Islam et al., 2023; Lamela & Figueiredo, 2018; Madigan et al., 2019). This cycle of neglect can have significant societal and economic costs, including increased healthcare utilization, social service involvement, and criminal justice involvement (World Health Organization, 2020). Besides, studies have shown that a parent's experience of child maltreatment increases the probability of engaging in abusive behavior toward their child (Armfield et al., 2021; Capaldi et al., 2019; Widom et al., 2015; Yang et al., 2018). Some earlier empirical studies indicate a relationship between grandparental investment and mental health problems in their grandchildren (Jappens & Van Bavel, 2020; Sadruddin et al., 2019), while Tanskanen and Danielsbacka (2018) argue that the relationship is not necessarily causal (Helle et al., 2022). In this context, several researchers have proposed conceptual mechanisms to explain the relationship that grandparents exert with their grandchildren and investigate the continuation and discontinuation of the intergenerational transmission of child maltreatment, such as Dixon et al. (2009), Egeland et al. (1988), Islam et al. (2023), Jaffee et al. (2013), and Mckenzie et al. (2021)).

In this regard, Islam et al. (2023) analyze mental health outcomes across grandchildren ages 8 to 38 and test the additive risk hypothesis with others. Despite their extensive analysis, they found no supportive evidence for the additive risk hypothesis, indicating that combined generational maltreatment did not worsen mental health outcomes in grandchildren. In contrast, our study distinctively examines these effects specifically in older adults, focusing on grandchildren who are 40 years of age and above. This specific age focus allows us to explore the longer-term, additive impacts of familial maltreatment in those demographic areas where such histories could have quite distinct presentations of consequences. Our study also revisits the intergenerational impact of child maltreatment. However, it goes a step further to extend the investigation by applying structural equation modeling, which enables the dissection of the mediating roles of parental and grandparental influences, thus affording a nuanced understanding of how maltreatment is transmitted across generations.

Understanding how grandparental neglect can affect the mental health of their grandchildren requires a theoretical explanation of how maltreatment can be transmitted across generations. Social learning theory is fundamental for understanding the transmission of maltreatment, particularly physical maltreatment and harsh parenting (Bandura, 1973). According to this theory, parents are alleged to repeat the parenting practices of their own parents due to their misperceived “positive effects” (Alink et al., 2019). They normalize the use of physical maltreatment as a form of discipline by modeling the physical neglect perpetrated by their own parents (Erten & Keskin, 2020). According to research (Badenes-Ribera et al., 2020; Yang et al., 2018), physical abuse increases the risk of subsequent harsh parenting or the physical abuse of one's own children. Another important channel to consider is the effect of grandparental neglect on the attachment styles of their children, who are the parents of the grandchildren. Attachment theory explains how parent-child relationships develop and shape children's character (Bowlby, 1978). Maltreated children have insecure or disorganized attachments compared to those of nonmaltreated children, and insecure or unresolved adult attachments are related to subsequent parenting problems and maltreatment behaviors (Cyr et al., 2010; Marshall et al., 2022; Reijman et al., 2017).

Consequently, one can identify that its potential effects may differ between maternal and paternal grandparents during the study of intergenerational transmission of child neglect in the aspect of attachment theory (Crocetto, 2019; Yehuda & Lehrner, 2018). Some studies suggest that the influence of grandparents on grandchildren may be greater for the maternal compared to the paternal grandparents. This is possible because, based on traditional family relationships, mothers are expected to have a more frequent and involved caring role (Chan & Elder, 2000; Coall & Hertwig, 2010). However, it is a complex, multidimensional phenomenon that requires further exploration, underlining the necessity to differentiate between maternal and paternal grandparents.

By examining the effect of maternal and paternal grandparents separately, we aim to expand upon the current literature and investigate the following research questions: *To what extent does grandparental child neglect in the first generation predict the probability of mental health problems in the third generation, and whether neglectful maternal and paternal grandparents have differential effects on their grandchildren?*

## 2 Data

Our study uses individual-level data from the Tromsø Study,^2^ a cohort study involving the residents of Tromsø, the largest city in Northern Norway with approximately 77,000 inhabitants. From 1974 to 2016, the Tromsø Study, also known as Tromsø 1–7, has been conducted in seven waves, with participation rates ranging from 64.7% to 78.5% (Sari et al., 2023). The survey data includes health-related information on the adult population residing in Tromsø and is representative of the overall adult population in Norway, as indicated by previous studies (Olsen et al., 2020).^3^

**Fig. 1:**
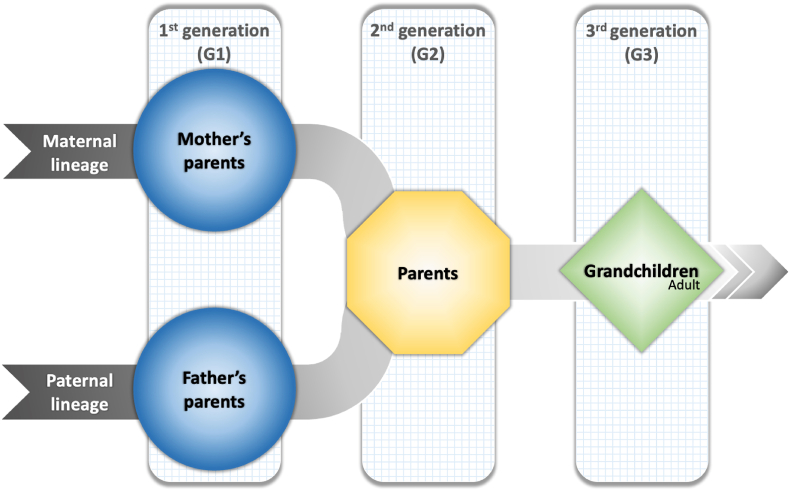
Definition and presentation of generations. *Note*: This diagram starts with the first generation (G1), consisting of the maternal and paternal grandparents, and proceeds to the third generation (G3), the adult grandchildren. G1 is not a participant in the Tromsø Study but is represented by reports from Generation 2 (G2), their children. G2, the parents of G3, report on their experiences of neglect by G1. G3, all adults over age 40, provide their own reports of neglect by G2 and depression.

Specifically, we specifically used data from the seventh wave of the Tromsø Study (Tromsø7), conducted between 2015 and 2016.^4^ The reason for selecting this particular study was the presence of the child neglect variable in the data set. In line with Sari et al. (2023), using family ID numbers provided by the Norwegian Tax Administration, we were able to establish family connections between participants in the Tromsø Study for the first time within the context of this study. Our sample has 1361 observations in total who completed the relevant survey questions, including participants from two generations, grandchildren (G3) and their parents (G2). Information regarding the maternal and paternal grandparents (G1) was gathered through responses from the G2 participants. Fig. 1 presents the definitions of generations and demonstrates the linkages between family members across generations.

### 2.1 Measures

*Dependent variable*: depression. — We assessed the mental health status of the G3 using self-report measures of depression as a proxy. To trace the mental health of G3, we consider the respondents’ answers to the question, “Have you felt depressed or sad during the last week?“. To facilitate data analysis, we dichotomized responses following previous research (Byrow et al., 2022; Kim et al., 2021). Responses indicating “no complaint” were used as a reference point, while those indicating “little,” “pretty much,” or “very much” complaints were considered indicative of depression in G3. With this classification, depression was reported by 29.7% of participants in our study sample (Table 1).

**Table 1:** Study sample characteristics.

Variable names	N	% (Median)	Standard deviation	Min.	Max.
*Third generation (G3) – Grandchildren*					
Mental health status					
Not depressed (ref)	957	70.3			
Depressed	404	29.7			
Gender					
Female (ref)	685	50.3			
Male	676	49.7			
Marital status^a^					
Single (ref)	642	47.2			
Married	719	52.8			
Year of birth^b^	1361	1969	4.77	1951	1975
Household income					
Lower (ref)	247	18.1			
Higher	1114	81.9			
*Second generation (G2) – Parents*					
Child-neglecting parents					
No (ref)	1295	95.2			
Yes	66	4.8			
Household economic status^c^					
Lower (ref)	165	12.1			
Higher	1196	87.9			
*First generation (G1) - Grandparents*					
Maternal child-neglecting grandparents^d^					
No (ref)	1209	90.8			
Yes	123	9.2			
Paternal child-neglecting grandparents^d^					
No (ref)	1214	94.1			
Yes	76	5.9			
Household economic status^c^					
Lower (ref)	655	48.1			
Higher	706	51.9			

Note.

a Marital status is divided into two categories: “single”, which includes persons who are single, widowed, divorced, or separated, and “married”, which includes persons in registered partnerships.

b The median of the year of birth variables for individuals in G3 and G2 is reported.

c To classify the household economic status of G2 raising G3 and G1 raising G2, two categories were created based on responses to the question, “How was your family's financial situation during childhood?” Responses indicating “difficult” and “very difficult” were grouped under lower household economic status, while responses indicating “good” and “very good” were grouped under higher household economic status.

d Child-neglecting grandparents are at least maternal or paternal grandparents who neglected their child (G2) while raising them. The shares of child-neglecting grandparents are presented separately for maternal and paternal grandparents (G1). These variables in our sample have some missing observations, but the proportion of missing data is trivially low. To address this issue, we used the listwise deletion method (Conde & Poston, 2020; Hoover & Perez, 2004).

*Covariates*. — Child neglect in this context refers to parents/caregivers who did not provide their children with the basic necessities of food, clothing, shelter, and care/love.^5^ The child neglect variable for grandparents referred to the failure of G1 parents to provide adequate care for their children (G2); the same applied to the failure of G2 parents to provide adequate care for their children (G3). Among the G2s in our sample, 4.8% neglected their G3 child (Table 1). Among G1s, 9.2% of maternal G1s and 5.9% of paternal G1s were neglectful.^6^

We also control for several individual-level demographic and socioeconomic status variables for G3, G2, and G1, as shown in Table 1. Household economic status is used as a proxy for socioeconomic status, and controls for the gender, year of birth, and marital status of G3 individuals are included (Berlin et al., 2011; Haugland et al., 2021; Kong et al., 2021; Langevin et al., 2023). Furthermore, we include the total taxable household income^7^ for G3 individuals in the year prior to their participation in the study (Lamela & Figueiredo, 2018). The responses were divided into two groups, which is consistent with Statistics Norway's household income and wealth statistics (Statistics Norway, 2022). Those who earned 551,000–750,000 Norwegian kroner or more were classified as the higher income group, while the rest were classified as the lower income group, which serves as a reference. The household economic status of G2 and G1 individuals during upbringing is also included (Maniar et al., 2019) and grouped into lower and higher categories, with the lower as the reference group. We reported the correlation matrix for the binary variables in Table 2.

**Table 2:** – Correlation matrix.

Variables	(1)	(2)	(3)	(4)	(5)	(6)	(7)	(8)	(9)
G3 Mental health status	1.000								
G3 Gender	−0.031	1.000							
G3 Marital status	−0.053	0.023	1.000						
G3 Household income	−0.108	0.089	0.384	1.000					
G2 Child-neglect	0.111	−0.020	−0.049	0.003	1.000				
G2 Household economic status	−0.045	−0.054	0.035	0.006	−0.130	1.000			
Maternal G1 Child-neglect	0.023	−0.047	−0.020	−0.020	0.128	−0.077	1.000		
Paternal G1 Child-neglect	0.028	0.005	−0.016	−0.019	0.028	−0.060	0.022	1.000	
G1 Household economic status	0.021	−0.029	−0.026	−0.043	0.029	−0.100	0.087	0.071	1.000

*Note*: This table presents the correlation coefficients among the key binary variables used in the study, including mental health status, gender, marital status, household income, child neglect, and household economic status across three generations (G1, G2, and G3). The Phi coefficient was used to calculate the correlations between these binary variables, which provides a measure of the strength and direction of the association between two binary variables. Correlations closer to 1 or -1 indicate a stronger relationship, while correlations closer to 0 indicate a weaker or no relationship. Year of birth was excluded from this matrix as it is a continuous variable and does not fit within the binary correlation structure used here.

## 3 Empirical strategy

We apply a comprehensive analytical framework that tests how the relationships between G1 and G2 child neglect are related to G3 mental health status. Using linear probability and structural equation models, we explain the mechanism underlying the effect and the processes through which G3 mental health outcomes are influenced.

### 3.1 Linear probability model with ordinary least squares (OLS)

We use a linear probability model to estimate these associations for the mental health status of G3 using ordinary least squares regression. In support of our econometric approach, previous research conducted by Hellevik (2009) has shown the utility of linear regression in modeling binary dependent variables. The advantage of our approach is that it produces coefficients and proportional differences that can be interpreted as the change in probability of a specific value of the dependent variable while holding all other independent variables constant (Wooldridge, 2010). However, one problem of linear probability models related to heteroscedasticity is noted by Hellevik (2009) and Mood (2010). In order to address this problem, we use heteroscedasticity-robust standard errors.

Our empirical model aims to estimate the probability of a relationship between G3 mental health status, specifically depression, and both G1 and G2 child neglect, defined as the failure of parents/caregivers to provide adequate care for their children. The equation used for this estimation is as follows:

$$Y_{G3}=\beta_{0}+\beta_{1}S_{G1}+\beta_{2}S_{G2}+\beta_{3}S_{G1}\times S_{G2}+\beta_{4}C+\varepsilon \text{,}$$

where $Y_{G3}$ is a binary variable that equals one if G3 reported depression and zero otherwise. As mentioned, G3 represents the adult grandchild generation, G2 represents the parent generation, and G1 represents the grandparent generation. $S_{G2}$ is a binary variable that equals to one if at least one of the G2 parents/caregivers neglected G3 during their upbringing, and zero otherwise. Similarly, $S_{G1}$ is a binary variable that equals to one if at least one of the G1 parents/caregivers neglected G2 during their upbringing, and zero otherwise. C is the vector of the control variables, which include gender, marital status, and year of birth for the G3 generation. $\beta_{0}$ is the intercept; $\beta_{1}$ and $\beta_{2}$ are the coefficients on the main explanatory variables, G1 child neglect (which indicates whether any maternal or paternal grandparent neglected G2 during their upbringing) and G2 child neglect, respectively. The estimated coefficient $\beta_{2}$ can be interpreted directly as the change in the probability of a grandchild having poorer mental health due to the parents' child neglect. The interaction term $S_{G1}\times S_{G2}$ in our model captures the joint effect, moderation, of G1 and G2 child neglect on G3 mental health problems, testing the additive risk hypothesis. The coefficient $\beta_{3}$ measures the change in the probability of G3 mental health problems associated with the interaction between G1 and G2 child neglect. A positive and statistically significant $\beta_{3}$ would indicate that the effect of grandparental neglect on G3 mental health is greater when parental neglect is also present, possibly indicating that parental neglect amplifies the adverse effects of grandparental neglect on G3 mental health. In addition, we include the separate variables for maternal and paternal G1 child neglect in our model to explore whether the effect on G3 mental health differs based on the parental side, as the lineage of grandparents. $\beta_{4}$ represents the coefficients associated with the control variables. For G2, it includes the household economic status during the period when G3 was being raised. Similarly, for G1, it includes the economic conditions of the household during the upbringing of G2. ε is the error term.

To validate the reliability of our findings, we also conduct probit regressions using the same set of variables as our main model to estimate the average marginal effect (Wooldridge, 2010). Our analysis shows that the average marginal effects of the probit model closely matched the OLS coefficients, indicating that the results are robust to changes in the regression method used.

### 3.2 Mediation analysis

**Fig. 2:**
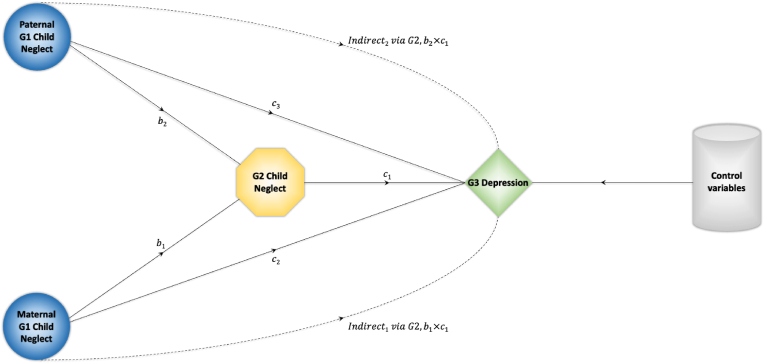
Conceptual framework of Structural Equation Modeling.

In our study, we employ Structural Equation Modeling (SEM) to analyze the pathways through which G1 and G2 child neglect influence the mental health of adult G3, explicitly focusing on the presence of depression (see Fig. 2). Our SEM approach enables us to explore not only the direct impacts but also the mediating role of parental neglect on the effects of grandparental neglect. Thus, we test sequential mediation pathways examining the independent and cascading effects of both maternal and paternal grandparents' history of child neglect and parental child neglect on the adult grandchild's mental health. We anticipate that the effect of G1 neglect on G3's mental health could be worsened by neglect from G2. Through mediation analysis, we test this hypothesis by evaluating whether the path from G1 to G3, via G2, amplifies the risk, suggesting a compounding rather than merely additive effect of neglect across generations.

Our model treats maternal and paternal G1 child neglect as distinct exogenous variables that potentially influence G2 child neglect, which, in turn, is assessed for its direct and mediated effect on G3's depression. As in the previous analyses, we control for the same variables, accounting for additional variance in the depression outcomes of G3. Our comprehensive approach presents the transmission mechanisms that continue across generations, which may not be evident through direct effects alone. We evaluate the model fit by using several criteria to ensure robustness: a $\chi^{2}/df$ ratio less than 5, Root Mean Square Error of Approximation (RMSEA) below 0.06, Standardized Root Mean Square Residual (SRMR) under 0.08, and Comparative Fit Index (CFI) above 0.95. These results align with established standards in SEM (Russotti et al., 2021; Schermelleh-Engel et al., 2003) and confirm that our model effectively captures the complex dynamics within the data. Indirect effects are further investigated through bootstrapping with 10,000 replications, providing confidence intervals that statistically validate the mediation paths delineated in our model.

## 4 Results

Table 3 presents estimation results testing intergenerational transmission of child neglect to mental health in G3. Columns (1) and (2) report OLS regression, while columns (3) and (4) present probit marginal effects for G3's mental health status. The G1 child neglect variable represents whether any grandparent (either maternal or paternal) neglected their children (G2). This aggregate measure is used to provide an overview of the general effects before we further analyze maternal and paternal grandparent neglect separately in Table 4.

Results in Table 3 clearly indicate that there is a significant positive association between G2 child neglect and the mental health status of their children (G3), holding both in OLS and in the Probit models. The estimated coefficients for G2 child neglect in the OLS and the average marginal effect in the probit model were 0.202 (Table 3, column (1)) and 0.204 (Table 3, column (3)), respectively. These results show a significant association between parental neglect in G2 and probability of depression in G3. It suggests that children in G3 are more likely to experience mental health challenges when their parents (G2) display neglectful behaviors. However, our findings present no significant relationship between G1's neglectful behavior toward their children (G2) and the mental health outcomes of their grandchildren (G3), which probably indicates that the effects of G1's neglect are not likely to influence their grandchildren's mental health.

We further look at the interaction between G1 child neglect and G2 child neglect to examine whether the presence of G2 child neglect amplifies the effect of G1 child neglect on G3 poorer mental health (Table 3, columns (2) and (4)). As in Islam et al. (2023), this interaction term is not statistically significant, suggesting that the effect of G1 child neglect on G3 mental health is not moderated by the presence or absence of G2 child neglect. It is important to note, however, that the absence of a significant interaction effect does not necessarily mean that G1 child neglect has no effect on G3 mental health status. To further investigate the relationship between G1 child neglect and G3 mental health, our model distinguishes between maternal and paternal grandparent neglect, as detailed in Table 4.

**Table 3:** Results of the effect of neglect from grandparents and parents on grandchildren's mental health.

Variables	Dependent variable: *Mental health status of G3*			
	OLS		Probit (Marginal effects)	
	(1)	(2)	(3)	(4)
G2 Child-neglect	0.202∗∗∗	0.200∗∗∗	0.204∗∗∗	0.202∗∗∗
	(0.064)	(0.083)	(0.065)	(0.084)
G1 Child-neglect	−0.008	−0.008	−0.011	−0.011
	(0.036)	(0.037)	(0.037)	(0.039)
G1 Child-neglect x G2 Child-neglect		0.004		0.005
		(0.128)		(0.115)

Control variables	^*✓*^	^*✓*^	^*✓*^	^*✓*^
Observations	1361	1361	1361	1361
*R-squared*	0.027	0.027		
*AIC*			1638.6	1640.6

Note: Columns (1) and (2) present coefficients from OLS regressions, while columns (3) and (4) present marginal effects from probit regressions. The interaction between G1 child neglect and G2 child neglect is reported in columns (2) and (4). We have adjusted all estimates for G3's gender, year of birth, marital status, household income, and the economic status of both G2 and G3 households during their children's upbringing. The results for these control variables are presented in Appendix Table A.1 for OLS results, and Appendix Table A.2 for probit regressions. We assessed G3's mental health status using self-reported measures of depression. Heteroskedasticity-robust standard errors are shown in parentheses for OLS models, while delta method standard errors are shown in parentheses for probit models. AIC is Akaike Information Criterion.

∗∗∗ Significant at the 1% level.

∗∗ Significant at the 5% level.

∗ Significant at the 10% level.

**Table 4:** Results of the effect of child neglect from maternal and paternal grandparents and parents on grandchildren's mental health.

Variables	Dependent variable: *Mental health status of G3*			
	OLS		Probit (Marginal effects)	
	(1)	(2)	(3)	(4)
G2 Child-neglect	0.249∗∗∗	0.199∗∗∗	0.251∗∗∗	0.201∗∗∗
	(0.075)	(0.083)	(0.076)	(0.084)
Maternal G1 Child-neglect	−0.004	−0.052	−0.009	−0.057
	(0.062)	(0.065)	(0.067)	(0.068)
Paternal G1 Child-neglect	0.039	0.042	0.037	0.040
	(0.070)	(0.072)	(0.073)	(0.075)
Maternal G1 Child-neglect x G2 Child-neglect		0.379∗∗		0.426∗
		(0.180)		(0.222)
Paternal G1 Child-neglect x G2 Child-neglect		−0.059		−0.053
		(0.324)		(0.293)

Control variables	^*✓*^	^*✓*^	^*✓*^	^*✓*^
Observations	1258	1258	1258	1258
*R-squared*	0.024	0.027		
*AIC*			1517.6	1518.2

Note: Columns (1) and (2) present coefficients from OLS regressions, while columns (3) and (4) present marginal effects from probit regressions. The interaction between maternal and paternal G1s child neglect and G2 child neglect are reported in columns (2) and (4). We have controlled G3's gender, year of birth, marital status, household income, and the economic status of both G2 and G3 households during their children's upbringing. The results for these control variables are presented in Appendix Table A.3. We assessed G3's mental health status using self-reported measures of depression. Heteroskedasticity-robust standard errors are shown in parentheses for OLS models, while delta method standard errors are shown in parentheses for probit models. AIC is Akaike Information Criterion.

∗∗∗ Significant at 1% level.

∗∗ Significant at 5% level.

∗ Significant at 10% level.

Table 4 shows how neglect from both maternal and paternal grandparents correlates with mental health outcomes in grandchildren, offering insights into the patterns of intergenerational transmission of neglect. Similar to the results in Table 3, G2 child neglect still has a positive and statistically significant association with poorer offspring (G3) mental health in all specifications. Maternal G1 child neglect and paternal G1 child neglect do not show a statistically significant direct relationship with G3 mental health problems. However, the interaction variables between maternal and paternal G1 child neglect and G2 child neglect produce interesting results (Table 4). The positive coefficient value for the interaction between maternal G1 child neglect and G2 child neglect indicates that the adverse effect of maternal G1 child neglect on G3 mental health is stronger when combined with child neglect experienced by G2. Thus, the effect of childhood maltreatment on G3, together with neglectful maternal G1, is worse on offspring's mental problems than the effect of childhood maltreatment by parents (G2) alone. We do not find this effect for paternal grandparents. In summary, the results show that grandparents' effects on their grandchildren's mental health differ depending on whether they are maternal or paternal G1s.

We further explored the degree to which the economic status of households in both G2 and during the upbringing of their children further mediated this association between G2 child neglect and G3 mental health (see Appendix Table A.4). These findings show that G2 child neglect is still significantly associated with poor mental health of G3 even controlling for childhood economic conditions. Nevertheless, the interaction terms that included G2 child neglect and G2 household economic status, as well as G1 child neglect, were not statistically significant. This included the interaction terms for paternal G1 child neglect with G2 household economic status, maternal G1 child neglect with G2 household economic status, and G1 child neglect with G1 household economic status. Consequentially, the results concerning the interaction term suggest that economic factors do not significantly moderate the impact of neglect across generations in this sample.

**Fig. 3:**
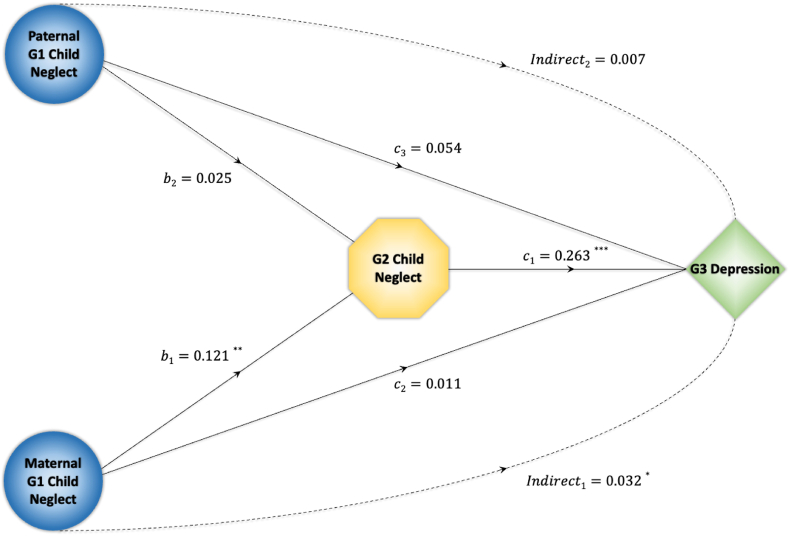
Indirect effects of maternal and paternal grandparents' child neglect on adult grandchildren's mental health. *Note*: Figure depicts the mediated effects of neglect by maternal and paternal grandparents (G1) on the mental health of adult grandchildren (G3), with parental (G2) neglect serving as the mediating factor. Solid lines represent direct effects, while dashed lines represent indirect effects through G2. Significance levels are denoted as follows: ∗p < 0.1; ∗∗p < 0.05; ∗∗∗p < 0.01.

Following the moderation results, our SEM analysis extends beyond correlations to present the direct and mediated pathways through which G1 and G2 neglect affects the subsequent generation's (G3) mental health outcomes, specifically focusing on depression in adulthood. As presented in Fig. 3, the indirect effects present how G1 child neglect transmits its impact through G2 to G3. The results show that the indirect effect of maternal G1 neglect is estimated at 0.032, indicating a significant increase in risk through maternal lineage neglect. This pathway represents the cumulative negative consequences that occur when neglect is passed down from maternal grandparents to parents. Conversely, the indirect effect via paternal G1 neglect is smaller and not statistically significant, indicating variability in transmission based on the lineage of the neglecting grandparent. The results also present the direct effect of G2 child neglect on G3 depression, with a coefficient of 0.263, indicating a significant influence.

## 5 Discussion

Child neglect is a pervasive problem with severe implications for the well-being of individuals and society as a whole. Our study aims to examine the association between the intergenerational transmission of child neglect and adult mental health outcomes. Our results suggest that the effect of childhood maltreatment by both neglectful maternal grandparents and parents is worse for the mental health of the grandchildren than the effect of childhood maltreatment by parents alone.

Our study contributes significantly to the literature on the intergenerational transmission of child neglect by taking a comprehensive approach to examine the sub-mechanisms perpetuating child maltreatment across generations. Here, we begin with the role of grandparents in the formation of a mental health legacy for future generations; we move beyond previous studies by introducing grandparents from both lines into the intergenerational transmission model from parent to child. The empirical evidence confirms the additive risk hypothesis, explaining how exposure to child neglect negatively affects the subsequent generation. The negative effects of child neglect are amplified across multiple generations by the repetition of child neglect in each generation. This finding differs from the findings of Islam et al. (2023).

Enlow et al. (2018), Langevin et al. (2023), and Noll et al. (2009) are studied the intergenerational continuity of cumulative child maltreatment and present negative effects on the offspring's mental health. Langevin et al. (2023) present that maternal emotional dysregulation and mother-to-child attachment are major contributors to such continuity. They address the need for additional support to be provided to multi-violence-affected families. According to Enlow et al. (2018), harmful environmental influence exposure, such as maltreatment of the offspring, can contribute to the development of physiological and neurocognitive vulnerabilities that form a poor mental health trajectory. Noll et al. (2009) emphasize the burden that children born into adversity are required to bear and compare the magnitude of this burden across demographically similar groups of individuals who differ in the presence of maternal childhood sexual abuse. Similarly, Erel et al. (2024) find that maternal psychopathology negatively influences child emotion regulation through maternal expressiveness, which in turn can exacerbate externalizing problems in children.

Our finding of the intergenerational transmission of child neglect between grandparents and parents is consistent with previous research on the intergenerational transmission of child neglect, which has demonstrated that children who experience neglect are more likely to repeat the cycle of neglect as adults (Bartlett et al., 2017; Berlin et al., 2011; Islam et al., 2023; Madigan et al., 2019). These studies mainly found that the possibly neglecting grandparents may be influencing childrearing practices of their own children, who might then reproduce similar parenting styles to their children, thereby perpetuating a history of neglect across generations. This assumption is based on previous literature showing that parenting styles are parental experience-dependent and that such experiences are passed on to subsequent generations (Capaldi et al., 2019; Seteanu & Giosan, 2022).

The intergenerational transmission of child neglect is further theoretically anticipated, in which a person experiencing neglect during his or her own childhood will increase the tendency for a repetition of this cycle in adulthood. Social learning theory states that children will learn from what they observe their parents and grandparents do. As pointed out by Kong et al. (2021), undergoing the experience enhances one's probability of re-enacting certain negative behaviors in the next generations. With this assumption, insecure attachment styles that result from careless and thoughtless parenting tend to increase the potential mental health problems in children (Schore, 2001). Otherwise, child neglect can raise attachment issues and parental-child relationship disruptions. In these ways, disrupted attachment relationships between parents and children could increase the risk of depression. Attachment theory is oriented toward one-directional emotional bonds between parents and children. For that matter, the children who are neglected do not have a secure attachment with their caregivers. This can create many negative effects, such as poor emotional regulation and relations in adulthood, according to Marshall et al. (2022). These negative effects may further be passed down to the next generation (Robboy & Anderson, 2011), as children of parents who are dismissive of attachment may have problems bonding with their children.

Additionally, genetic predispositions cannot be overlooked, along with the environment, in intergenerational child neglect. For example, Ahmadzadeh et al. (2023) point out that the genetic effects of anxiety and depression of mothers may have some effects on the temperament of children and perhaps later contribute to passing the pattern of neglect across generations. Also, Vinberg et al. (2024) discuss how childhood maltreatment affects neurotransmitter systems such as serotonin and dopamine, which are crucial for mood regulation and can be genetically modulated. These findings indicate that genetic factors can predispose individuals to both psychiatric conditions and behaviors that contribute to neglectful parenting, thus perpetuating the cycle of neglect across generations.

Grandparents and grandchildren can also be distinguished in terms of the sex of either the grandchild or the grandparent (Yehuda & Lehrner, 2018). Demographic changes and historical family structures have a profound effect on family dynamics and intergenerational transmission. Although maternal grandparents may have a significant effect on grandchildren, the absence of a similar effect for paternal grandparents does not necessarily mean there is no intergenerational transmission through the paternal side. Considering the traditional gender roles of the family in mid-1900s Norway, we know that mothers were primarily responsible for childcare (Lorentzen, 2013), which may contribute to the observed differences between maternal and paternal grandparents in the transmission of values and behaviors to the next generation. An evolutionary explanation is also at the forefront of theories that consider this phenomenon (Coall & Hertwig, 2010; Yehuda & Lehrner, 2018). In particular, maternal grandmothers are more certain of their biological ties to their grandchildren than other grandparents and make a more significant investment in their grandchildren's lives. Maternal grandparents who neglect their children may pass on this parenting style and behavior to their children, who in turn may be more likely to neglect their children, including their grandchildren.

### 5.1 Policy implications

To prevent the long-lasting and harmful effects of neglect on children's mental health, policymakers should prioritize implementing support programs focused on parents with inadequate parenting. These can involve parental education or training in empowering parents toward healthy attachment and positive parenting behaviors. Moreover, policymakers should consider improving access to mental health services, particularly for those who have experienced neglect or have a family history of neglectful parenting. This could be in the form of funding or other incentives directed towards mental services and community-based programs so that mental health providers specialize in treating persons who experience childhood neglect. Such interventions would, therefore, not only improve mental health among those who suffer from neglect but would also bring clarity into the cycle of neglectful parenting and yield better results at the family and societal levels.

### 5.2 Limitations and future directions

This study adds to the research on the intergenerational transmission of child neglect, but there are limitations that must be considered. Our study relies on self-report data that can create recall and social desirability biases. Future studies could also include observational data to augment their findings' validity. Our study is set within Norway alone; hence, generalization might not be the case in other countries. Therefore, future studies should further examine this intergenerational transmission of neglect among different populations to generalize the findings across different cultural backgrounds. Furthermore, unlike Duarte et al.'s (2016), our data are not distinguished by the possibility that at some point during childhood, one of the grandparents may co-reside with the offspring.

One potential limitation of our current design is the challenge of establishing causality, as it is uncertain whether poorer mental health biases the individual's perception of their parents or if the actual lack of care contributes to the development of depression. As pointed out by Islam et al. (2023), there is a possibility that children who have a predisposition to psychopathology may be more likely to experience negative parenting behaviors, such as abuse and neglect, rather than the other way around. Another limitation is that the measure used to assess parent-child relationships may reflect perceived rather than actual relationships, which may influence the participants' illness. Nonetheless, Koszycki et al. (2010) note that the perceived characteristics of parents hold significance in the development of psychological disorders and should not be disregarded.

Several directions for future studies can be built on our study and hold significant potential for advancing our understanding of the intergenerational transmission of child neglect. Future studies could thus test genetic mechanisms underlying this transmission, providing critical insights into the role of genetic factors in perpetuating child neglect across generations. It is important to explore potential moderating variables that could influence this transmission, such as the quality of the grandparent-grandchild relationship, the type and severity of maltreatment experienced in childhood by grandparents, and the cultural and social contexts. Besides, longitudinal studies are needed to examine the long-term effects of grandparents' neglect on their grandchildren's mental health outcomes and to determine the critical periods for intervention. Furthermore, future research should aim to include more comprehensive psychometric assessments to enhance the validity of the depression measurement. Additionally, categorizing the parent's generation into maternal and paternal neglect can yield more comprehensive insights into nuclear family dynamics. Lastly, control for childhood trauma impacts, such as early parental death, is valuable, as demonstrated by Böckerman et al. (2023).

## 6 Conclusion

Our study contributes to the existing research on the intergenerational transmission of child neglect by investigating the relationship between the child neglect of grandparents and the depression status of their adult grandchildren. The study findings suggest that the probability of depression in grandchildren is heightened when both their maternal grandparents and parents have neglectful parenting behaviors. Our study provides insight into a possible mechanism underlying this transmission of child neglect and evidence for additive risk hypotheses. It also fits into the theoretical framework in the intergenerational transmission of child neglect, pointing out the role of parenting styles and attachment in transmission. Our research underlines the necessity of interventions to break the intergenerational transmission cycle and promote future generations' mental health.

## Ethical statement

Regional Committees for Medical and Health Research Ethics (REK) - Norway approved this study (No: 32135) in 2019.

## Declarations of interest

None.

## CRediT authorship contribution statement

**Emre Sarı:** Writing – review & editing, Writing – original draft, Visualization, Software, Project administration, Methodology, Funding acquisition, Formal analysis, Data curation, Conceptualization. **Mikko Moilanen:** Writing – review & editing, Writing – original draft, Supervision, Data curation, Conceptualization. **Maarten Lindeboom:** Writing – review & editing, Writing – original draft, Methodology, Data curation, Conceptualization.

## Declaration of generative AI and AI-assisted technologies in the writing process

During the preparation of this work, the author(s) used Grammarly and OpenAI in order to improve the readability and language of the manuscript. After using this tool/service, the author(s) reviewed and edited the content as needed and take(s) full responsibility for the content of the publication.

## Data Availability

The authors do not have permission to share data.

## Footnotes

1 Child Protection Services Act (barnevernloven) of 17 July 1992 No. 100. The Child Protection Act ensures that children and youth who live in conditions that may harm their health and development receive the necessary help and care at the right time. It also ensures safe growing conditions for children and youth.

2 Regional Committees for Medical and Health Research Ethics approved the study, and informed consent was obtained from each participant prior to their enrollment (Sari et al., 2023).

3 Individuals with a university degree were slightly overrepresented in the study sample; however, overall, the population is regarded as typical of the Norwegian adult population as a whole (Olsen et al., 2020).

4 All residents of Tromsø municipality aged 40 and older were mailed an invitation, which included study information and access to complete the survey either on paper or online. For more details, https://uit.no/research/tromsostudy.

5 In the Tromsø7 study, participants were asked: “Failure of care in childhood, i.e. not having received the necessary of food, clothing, protection and care/love from parents/caregivers.” with the response options “yes” or “no."

6 In G1, we can determine the maternal and paternal grandparents based on the parents' gender. However, due to the limitations of the survey questions, which only inquire about parental neglect, it is not possible to distinguish between mothers and fathers in G2.

7 Total taxable income includes income from work, social benefits, and the like.
