# Supplementary material for: Long-term effects of grandparental child neglect on adult grandchildren's mental health: A three-generation study
Source: SSM Popul Health. 2024 Sep 24;28:101712. doi: 10.1016/j.ssmph.2024.101712 (PMC11471239; doi:10.1016/j.ssmph.2024.101712)
Supplement: Multimedia component 1 [file mmc1.docx]

Online Appendix

**Long-term Effects of Grandparental Child Neglect on Adult Grandchildren’s Mental Health:**

***A Three-Generation Study***

*by*

Emre Sarı,^1,2*^ Mikko Moilanen,^1^ Maarten Lindeboom^3^

School of Business and Economics, UiT The Arctic University of Norway, Tromsø, Norway.

^2^ Division for Health and Social Sciences, NORCE Norwegian Research Centre, Oslo, Norway.

^3^ School of Business and Economics, Vrije Universiteit Amsterdam, Amsterdam, Netherlands.

*Oslo, Norway*

September 2024

Table A.1 presents the results of OLS regressions examining the effect of neglect from grandparents on the mental health status of grandchildren, controlling for various demographic and economic factors. The dependent variable is the mental health status of G3 (grandchildren). The main independent variable of interest is G2 child neglect (neglect experienced by the parent of G3). The coefficients for G2 child neglect are positive and statistically significant at the 1% level across all eight specifications. The coefficient for G1 child neglect is not statistically significant in any of the eight specifications. This indicates that there is no evidence for the direct effect of grandparental neglect on the mental health of their grandchildren. Other control variables in the model include G3 gender, G3 year of birth, G3 marital status, G3 household income, G2 household economic status, G1 household economic status, and G1 child-neglect x G2 child-neglect interaction term. Among these, higher household income of G3 is negatively associated with the mental health of grandchildren, while being married to G3 is negatively associated with mental health in some specifications. Overall, the results suggest that the effect of neglectful parents in childhood has a significant association with the mental health of their children in adulthood. In contrast, the direct relationship between grandparental neglect and the mental health of their grandchildren is not significant.

**Table A.1 - Detailed results of the effect of neglect from grandparents and parents on grandchildren’s mental health: OLS regressions.**

|  | Dependent variable: *Mental health status of G3* | | | | |
| --- | --- | --- | --- | --- | --- |
|  | OLS | | | | |
| Variables | (1) | (2) | (3) | (4) | (5) |
| G2 Child-neglect | 0.210^***^ | 0.206^***^ | 0.212^***^ | 0.202^***^ | 0.200^**^ |
|  | (0.064) | (0.063) | (0.063) | (0.064) | (0.083) |
| G1 Child-neglect | 0.010 | 0.008 | 0.001 | -0.008 | -0.008 |
|  | (0.036) | (0.036) | (0.035) | (0.036) | (0.037) |
| G3 Gender |  | -0.028 | -0.020 | -0.021 | -0.021 |
|  |  | (0.025) | (0.025) | (0.025) | (0.025) |
| G3 Year of birth |  | -0.0001 | 0.001 | 0.001 | 0.001 |
|  |  | (0.003) | (0.003) | (0.003) | (0.003) |
| G3 Marital status |  | -0.051^**^ | -0.008 | -0.008 | -0.008 |
|  |  | (0.025) | (0.026) | (0.027) | (0.027) |
| G3 Household income |  |  | -0.141^***^ | -0.140^***^ | -0.140^***^ |
|  |  |  | (0.037) | (0.037) | (0.037) |
| G2 Household economic status |  |  |  | -0.042 | -0.042 |
|  |  |  |  | (0.040) | (0.040) |
| G1 Household economic status |  |  |  | 0.008 | 0.008 |
|  |  |  |  | (0.025) | (0.025) |
| G1 Child-neglect x G2 Child-neglect |  |  |  |  | 0.004 |
|  |  |  |  |  | (0.128) |
| Observations | 1,361 | 1,361 | 1,361 | 1,361 | 1,361 |
| *R-squared* | 0.010 | 0.014 | 0.026 | 0.027 | 0.027 |
| *Note*: All columns present coefficients from OLS regressions. Heteroskedasticity-robust standard errors are shown in parentheses for OLS models.  *** Significant at the 1% level.  ** Significant at the 5% level.  * Significant at the 10% level. | | | | | |

Table A.2 presents the detailed results on the intergenerational transmission of child neglect and the risk of mental health problems, exploring the effect of neglect from grandparents to grandchildren using probit regression to estimate the average marginal effects. The table presents the marginal effects for the dependent variable, mental health status of G3, with different independent variables, including G1 child neglect and G2 household economic status. Comparing Table A.1 and Table A.2, we can observe that the coefficients of the independent variables (G2 child neglect, G1 child neglect, G3 gender, etc.) are similar in both models. This indicates that both OLS and probit models provide similar estimates of the effect of these variables on the mental health status of G3. Overall, both OLS and probit models provide similar estimates of the effect of child neglect on the mental health status of grandchildren.

**Table A.2 - Detailed results of the effect of neglect from grandparents and parents on grand- children’s mental health: Probit regressions.**

|  | Dependent variable: *Mental health status of G3* | | | | |
| --- | --- | --- | --- | --- | --- |
|  | Probit (Marginal effects) | | | | |
| Variables | (1) | (2) | (3) | (4) | (5) |
| G2 Child-neglect | 0.210^***^ | 0.206^***^ | 0.215^***^ | 0.204^***^ | 0.202^**^ |
|  | (0.064) | (0.064) | (0.064) | (0.065) | (0.084) |
| G1 Child-neglect | 0.010 | 0.007 | -0.001 | -0.011 | -0.011 |
|  | (0.036) | (0.036) | (0.036) | (0.037) | (0.039) |
| G3 Gender |  | -0.028 | -0.021 | -0.023 | -0.023 |
|  |  | (0.025) | (0.025) | (0.025) | (0.025) |
| G3 Year of birth |  | 0.000 | 0.001 | 0.001 | 0.001 |
|  |  | (0.003) | (0.003) | (0.003) | (0.003) |
| G3 Marital status |  | -0.051^**^ | -0.008 | -0.008 | -0.008 |
|  |  | (0.025) | (0.027) | (0.027) | (0.027) |
| G3 Household income |  |  | -0.142^***^ | -0.140^***^ | -0.140^***^ |
|  |  |  | (0.038) | (0.038) | (0.038) |
| G2 Household economic status |  |  |  | -0.043 | -0.043 |
|  |  |  |  | (0.041) | (0.041) |
| G1 Household economic status |  |  |  | 0.008 | 0.008 |
|  |  |  |  | (0.025) | (0.025) |
| G1 Child-neglect x G2 Child-neglect |  |  |  |  | 0.005 |
|  |  |  |  |  | (0.115) |
| Observations | 1,361 | 1,361 | 1,361 | 1,361 | 1,361 |
| AIC | 1,648.763 | 1,649.229 | 1,635.892 | 1,638.569 | 1,640.567 |
| *Note*: All columns present marginal effects from probit regressions. Delta method standard errors are shown in parentheses. AIC is Akaike Information Criterion.  *** Significant at the 1% level.  ** Significant at the 5% level.  * Significant at the 10% level. | | | | | |

Table A.3 presents the findings of a regression analysis that investigates the effect of child neglect transmitted from maternal and paternal grandparents on the mental health status of grandchildren. The dependent variable is the mental health status of G3, and the independent variables include G2 child neglect and G1 child neglect by maternal and paternal grandparents. The table reports the coefficients for the OLS (columns (1)) and the marginal effects for the probit model (columns (2)). The results show that the child neglect experienced by G2 has a significant positive effect on the mental health problems of G3, regardless of whether the OLS or probit model is used. In contrast, the child neglect experienced by maternal and paternal grandparents does not have a significant effect on the mental health status of G3, as the coefficients are close to zero and not statistically significant.

Additionally, the table includes control variables such as gender, year of birth, marital status, household income, and household economic status for G2 and G1. Table A.3 also includes interaction terms between the child neglect experienced by G2 and that experienced by G1. The interaction term between maternal G1 child neglect and G2 child neglect has a significant positive effect on the mental health problems of G3. In contrast, the interaction term between paternal G1 child neglect and G2 child neglect is not significant. In general, the results of the OLS and probit models are quite similar, with both models indicating that G2 child neglect has a significant impact on the mental health status of G3 children.

**Table A.3 - Detailed result of the intergenerational transmission of child neglect and the risk of mental health problems: Exploring the effect of neglect from maternal and paternal grandparents on grandchildren.**

| Dependent variable: *Mental health status of G3* | | |
| --- | --- | --- |
|  | OLS | Probit (Marginal effects) |
| Variables | (2) | (4) |
| G2 Child-neglect | 0.199^**^ | 0.201^**^ |
|  | (0.083) | (0.084) |
| Paternal G1 Child-neglect | -0.052 | -0.057 |
|  | (0.065) | (0.068) |
| Maternal G1 Child-neglect | 0.042 | 0.040 |
|  | (0.072) | (0.075) |
| G3 Gender | -0.019 | -0.021 |
|  | (0.026) | (0.026) |
| G3 Year of birth | -0.00001 | 0.000 |
|  | (0.003) | (0.003) |
| G3 Marital status | -0.117^***^ | -0.117^***^ |
|  | (0.039) | (0.039) |
| G3 Household income | -0.009 | -0.009 |
|  | (0.028) | (0.028) |
| G2 Household economic status | -0.035 | -0.036 |
|  | (0.045) | (0.045) |
| G1 Household economic status | 0.007 | 0.007 |
|  | (0.026) | (0.026) |
| Maternal G1 Child-neglect x G2 Child-neglect | 0.379^**^ | 0.426^*^ |
|  | (0.180) | (0.222) |
| Paternal G1 Child-neglect x G2 Child-neglect | -0.059 | -0.053 |
|  | (0.324) | (0.293) |
| Observations | 1,258 | 1,258 |
| R2 | 0.027 |  |
| AIC |  | 1,518.2 |
| Note: Column (1) presents coefficients from OLS regressions, while column (2) presents marginal effects from probit regressions. Heteroskedasticity-robust standard errors are shown in parentheses for OLS models, while delta method standard errors are shown in parentheses for probit models. AIC is Akaike Information Criterion.  *** Significant at the 1% level.  ** Significant at the 5% level.  * Significant at the 10% level. | | |

Table A.4 examines the intergenerational transmission of child neglect and its relationship with G3's mental health, with a focus on potential mediation by economic conditions. The results indicate that G2 child neglect consistently has a significant and positive association with poorer mental health outcomes in G3, even when accounting for economic conditions. However, the interaction terms between G2 child neglect and G2 household status, as well as the interactions involving G1 child neglect, did not reach statistical significance in Table A.4. This contrasts with the results in Table 4 (main manuscript), where the interaction between maternal G1 child neglect and G2 child neglect was significant, suggesting a cumulative effect of maternal neglect across generations. The lack of significance in Table A.4’s interaction terms implies that economic conditions might not strongly moderate the impact of neglect across generations in this sample, and the previously observed interaction effect may be context-specific, possibly influenced by other unmeasured factors.

**Table A.4 – OLS results on the intergenerational transmission of child neglect and mental health risks, with mediation by childhood economic conditions**

|  | Dependent variable: *Mental health status of G3* | | | |
| --- | --- | --- | --- | --- |
|  | OLS | | | |
| Variables | (1) | (2) | (3) |  |
| G2 Child-neglect | 0.179^*^ | 0.248^***^ | 0.206^***^ |  |
|  | (0.104) | (0.075) | (0.063) |  |
| G2 Household economic status | -0.046 | -0.010 | -0.039 |  |
|  | (0.042) | (0.047) | (0.040) |  |
| Paternal G1 Child-neglect |  | 0.287 |  |  |
|  |  | (0.188) |  |  |
| Maternal G1 Child-neglect |  | 0.155 |  |  |
|  |  | (0.176) |  |  |
| G3 Gender | -0.021 | -0.019 | -0.021 |  |
|  | (0.025) | (0.026) | (0.025) |  |
| G3 Year of birth | 0.001 | -0.0001 | 0.001 |  |
|  | (0.003) | (0.003) | (0.003) |  |
| G3 Household income | -0.140^***^ | -0.120^***^ | -0.140^***^ |  |
|  | (0.037) | (0.039) | (0.037) |  |
| G3 Marital status | -0.007 | -0.009 | -0.009 |  |
|  | (0.027) | (0.028) | (0.027) |  |
| G1 Child-neglect | -0.007 |  | -0.085 |  |
|  | (0.036) |  | (0.057) |  |
| G1 Household economic status | 0.008 | 0.007 | -0.005 |  |
|  | (0.025) | (0.026) | (0.027) |  |
| G2 Child-neglect x G2 Household economic status | 0.036 |  |  |  |
|  | (0.131) |  |  |  |
| Paternal G1 Child-neglect x G2 Household economic status |  | -0.293 |  |  |
|  |  | (0.202) |  |  |
| Maternal G1 Child-neglect x G2 Household economic status |  | -0.191 |  |  |
|  |  | (0.187) |  |  |
| G1 Child-neglect x G1 Household economic status |  |  | 0.112 |  |
|  |  |  | (0.072) |  |
|  |  |  |  |  |
| Observations | 1,361 | 1,258 | 1,361 |  |
| R2 | 0.027 | 0.027 | 0.027 |  |
| Note: OLS regression results are presented under columns (1), (2), and (3) based on interaction variables. Heteroskedasticity-robust standard errors are shown in parentheses.  *** Significant at the 1% level.  ** Significant at the 5% level.  * Significant at the 10% level. | | | | |
